# Thyroid Carcinoma Coexisting with Hashimoto’s Thyreoiditis: Clinicopathological and Molecular Characteristics Clue up Pathogenesis

**DOI:** 10.1007/s12253-019-00580-w

**Published:** 2019-01-21

**Authors:** Csaba Molnár, Sarolta Molnár, Judit Bedekovics, Attila Mokánszki, Ferenc Győry, Endre Nagy, Gábor Méhes

**Affiliations:** 1 Department of Pathology, University of Debrecen, Nagyerdei krt. 98, Debrecen, H-4042 Hungary; 2 Department of Surgery, Clinical Center, University of Debrecen, Debrecen, Hungary; 3 Department of Internal Medicine, Clinical Center, University of Debrecen, Debrecen, Hungary

**Keywords:** Thyroid, Carcinoma, Hashimoto thyreoiditis, Clinicopathology, Molecular pathology

## Abstract

Thyroid cancer (TC) coexisting with Hashimoto’s thyroiditis (HT) presents with several characteristic features including multifocality and lower clinical stages compared to de novo carcinomas but its exact biology is still not understood. We reexamined clinico-pathological and molecular correlations between Hashimoto’s thyroditis and papillary thyroid cancer. A total of 262 patients with TC was evaluated who underwent thyroidectomy at the Surgical Department of the University of Debrecen. Clinical data, histology and molecular data were evaluated. Our cohort included 43 patients (16.4%) with (5 male, 38 female) and 219 (83.6%) patients without coexisting HT (48 male, 171 female). Hashimoto’s thyroiditis related thyroid cancer presented predominantly (93.0% of the cases) with the papillary histological type. Multifocality was observed more frequently with coexisting HT (16/40; 40.0%) compared to cases uninvolved (45/190; 23.7%)(*p* = 0.034). In contrast, lymphatic metastasis (pN1) with a significantly reduced frequency in patients with HT (4/11; 36.4%) then without HT (34/41 pN1; 82.9%)(*p* = 0.002). BRAF V600E mutation could be demonstrated at significantly lower rates in cases of PTC + HT (32.1 vs 60.7%, *p* < 0.005). High incidence, multifocality and papillary morphology strongly support a causal relation between TC and preexisting Hashimoto’s thyroiditis, the latter to be considered as a preneoplastic condition promoting thyroid carcinogenesis.

## Introduction

Hashimoto’s thyreoiditis (HT) is a frequent organ specific autoimmune disease affecting the thyroid gland, leading to the destruction of the glandular parenchyma and thyroid hypofunction with decreased T3 and T4 levels and subsequent elevation of TSH. The clinical diagnosis of HT is based on functional tests and the demonstration of specific autoantibodies. However, the consequences of the hyperergic immunological process representing parenchymal damage and lymphatic infiltrate is clearly represented in histological samples following surgery [1–3].

Thyroid cancer is one of the most common neoplasias of the endocrine system [4]. The vast majority (87.9%) takes the form of papillary thyroid carcinoma (PTC) [5], less frequently follicular (FTC), medullary (MTC) and anaplastic carcinomas (ATC) may occur. The diagnosis of the PTC is based on the histopathological examination of the thyroid mass detected by radiological imaging and aspiration cytology. In a significant portion of cases PTC develops in multiple areas of the thyroid parenchyma simultaneously, directing to a common etiological background. However, the only evidence-based etiologic factor in the pathogenesis of thyroid cancer to date is ionizing radiation [6–8] which only rarely occurs in the patient history. Further etiological correlations has been continously discussed. As such, earlier studies found that PTC is more common among patients who suffered from autoimmune lymphocytic thyroiditis [9–12], while others debated these findings [13–15]. The association between the two diseases was also supported by some more recent cross-sectional studies [16–29]. This potential relationship is generally explained by the misconducted follicular epithelial regeneration following chronic inflammatory damage, however, the molecular pathomechanism remains unclear. Cancer multifocality was also repeatedly presented in association with HT, providing a further basis for the causal relation between the two disorders [16, 17, 23, 30].

Molecular genetic analyses revealed several frequent DNA anomalies in thyroid cancer, including the mutations of BRAF [31], NRAS [32] and RET/PTC [33] genes, the prognostic significance of which is still debated in the individual subcategories of thyroid carcinoma [34]. The frequency of the BRAF mutation V600E was reported to be controversial in association with multiple cancer foci and HT involvement [35].

The aim of our current research was to examine the frequency and the clinico-pathological characteristics in the cases of coexisting TC and HT. We specifically focused on currently available factors associated with thyroid carcinogenesis, including histology, tumor multifocality and further, the prominent performers of the MAP kinase pathway, BRAF and NRAS mutational status.

## Materials and Methods

We re-examined the histological samples of patients who were operated due to thyroid disease at the Department of Surgery, University of Debrecen between 2007 and 2012. All cases with the final diagnosis of malignant thyroid disease were selected from the clinical database. Non-epithelial thyroid neoplasias were excluded from the further evaluation. The original histological samples with representative thyroid tissue were collected for systemic review of the thyroid cancer and the potential involvement by HT. The study population was thus formed by 262 samples with thyroid carcinoma.

Histological revision was made by an uniform evaluation scheme. The histological diagnosis of HT was stated if chronic lymphocytic infiltration, secondary lymphatic follicules and follicular atrophy could be recognized. These findings were occasionally extended by the additional presence of Hürthle cell metaplasia. The diagnosis of PTC was confirmed when the classical histological criteria were provided: characteristic cytoarchitecture, psammoma-bodies, nuclear abnormalities (enlarged nulcei, overlapping of nuclei, nuclear clearing, irregular nuclear membrane, nuclear grooves, pseudoinclusisons). In addition to the conventional H&E staining, immunohistochemistry was performed to detect the tissue antigens HBME1, galectin-3, CD56 and CK19 to support the identification of the PTC cell groups. The multifocal nature of the thyroid cancer has been stated when more than one foci were found in a distance of over 5 mm during the review of the complete surgical material. Individual foci were evaluated separately.

BRAF V600E mutation data were available in 185 cases demonstrated from the individual tumor foci selected by histology. DNA was extracted from unstained FFPE tissue material following macrodissection. PCR amplification and direct sequencing of BRAF exon 15 as well as NRAS exons 2, 3 and 4 was performed by the routine procedure of our Molecular Diagnostic Laboratory at the Department of Pathology, University of Debrecen. Sequencing was done using the ABI 310 genetic analyzer and Big Dye chemistry (Applied Biosystems, Foster City, CA, USA). In parallel, immunohistochemistry with the VE1 antibody (Roche Diagnostics, Mannheim, Germany) was applied in a routine session applying the Ventana Benchmark (Roche Diagnostics) immunohistochemistry station to demonstrate mutant BRAF protein in thyroid cancer cells in tissue sections.

The histological and clinicopathological data were statistically evaluated using t-test for the mean age, chi-square test for the male/female ratio, multifocality, lymph node involvement and lymph node dissection. The tumor and TNM stage were compared by Mann-Whitney test. BRAF mutation status in the selected case groups was compared by chi-square and Fischer-tests using the GraphPad Prism 6.04 Trial software (GraphPad Software, Inc. La Jolla, CA, USA).

## Results

### Patients and Basic Histology

Among the 262 thyroid carcinoma samples analyzed we found 43 cases (16.4%) with clear histological signs of HT involvement. The gender distribution showed a female predominance (38 female patients out of 43, 88.4%). The histological type of the thyroid carcinoma proved to be PTC in the majority of the cases (40 out of 43, 93.0%), the remaining 3 cases were FTCs (7.0%). In contrast, the male vs. female ratio was 48 vs. 171 in the 219 carcinoma patients without HT (78.1% female dominance). Within the latter group the histology resulted PTC in 190 cases (86.7%), FTC in 15 (6.9%), medullary carcinoma in 12 cases (5.5%) and anaplastic carcinoma in 2 cases (0.9%) (Table 1).

**Table 1:** Clinicopathological distribution of thyroid carcinomas with or without Hashimoto’s thyroiditis evaluated in the study (*n* = 262)

	**HT + thyroid cancer**	**Thyroid cancer without HT**	***p***
**Gender distribution**			
• All pts			
- Male/ all pts. (%)	5/43 (11.6%)	48/219 (21.9%)	
- Female/ all pts. (%)	38/43 (88.4%)	171/219 (78.1%)	ns
• PTC pts			
- Male/ all PTC pts. (%)	4/40 (10.0%)	26/190 (23,2%)	
- Female/ all PTC pts. (%)	36/40 (90.0%)	164/190 (76.8%)	0.046
• FTC pts			
- Male/ all FTC pts. (%)	1/3 (33.3%)	0/15 (0.0%)	
- Female/ all FTC pts. (%)	2/3 (66.7%)	15/15 (100%)	0.021
**Age of pts (years)**			
• Age of all pts	43.86 ± 16.29	48.78 ± 16.69	ns
• Age of PTC pts	44.03 ± 16.18	48.03 ± 16.74	ns
• Age of FTC pts	41.67 ± 21.55	54.93 ± 11.27	ns
**Histological subtype of tumors**			
• Papillary/ all cases (%)	40/43 (93.0%)	190/219 (86.7%)	
- Classic	28/40 (70.0%)	143/190 (75.3%)	
- Follicular variant	9/40 (22.5%)	34/190 (17.9%)	
- Other	3/40 (7.5%)	13/190 (6.8%)	
• Follicular/ all cases (%)	3/43 (7.0%)	15/219 (6.9%)	ns
• Medullary/ all cases (%)	0/43 (0.0%)	12/219 (5.5%)	ns
• Anaplastic/ all cases (%)	0/43 (0.0%)	2/219 (0.9%)	ns

Among PTC patients the proportion of the female gender was significantly higher in case of coexisting HT (36/40; 90.0%), than without HT (164/190; 76.8%)(*p* = 0.046). In the small group of FTC, the involvement of females also dominated: 2/3 (66.6%) FTCs with and all FTCs (15/15, 100%) without HT were female patients (*p* = 0.021). The mean age of PTC and FTC patients was somewhat lower for tumors with HT, than without, but these differences were not significant.

### Thyroid Cancer Multifocality and HT

The occurance of multiple cancer foci within the same resection specimen was also found to be significantly influenced by the presence of HT (Table 2). Multifocality of PTC was significantly more frequent with coexisting HT (16/40; 40.0%) compared to cases uninvolved (45/190; 23.7%)(*p* = 0.034). Multifocality of FTC, however, did not correlate with the presence of HT as none of the FTC patients with HT showed mutifocal tumor (0/3; 0%), while a minor group of patients without HT did (4/15; 26.7%)(*p* = 0.196).

**Table 2:** Intra-organ distribution and pathological stage of thyroid carcinomas with and without coexisting HT (MF = multifocal, UF = unifocal)

	**HT + TC**	**TC without HT**	***p value***
**Multifocality**			
• PTC			
- MF/ all PTC cases (%)	16/40 (40.0%)	45/190 (23.7%)	
- UF/ all PTC cases (%)	24/40 (60.0%)	145/190 (76.3%)	0.034
• FTC			
- MF/ all FTC cases (%)	0/3 (0.0%)	4/15 (26.7%)	
- UF/ all FTC cases (%)	3/3 (100.0%)	11/15 (73.3%)	ns
**pT tumor stage**			
• PTC	1.33 ± 0.73	1.48 ± 0.76	ns
• FTC	1.33 ± 0.58	1.92 ± 1.19	ns
**pN lymph node status**			
• PTC			
- pN1/ all PTC cases	4/11 (34.6%)	34/41 (82.9%)	
- pN0/ all PTC cases	7/11 (65.4%)	7/41 (17.0%)	0.002
• FTC			
- pN1/ all FTC cases	0/1 (0.0%)	1/1 (100.0%)	
- pN0/ all FTC cases	1/1 (100.0%)	0/1 (0.0%)	ns
**TNM stage**			
• PTC cases	1.20 ± 0.61	1.33 ± 0.79	ns
• FTC cases	1.00 ± 0	1.69 ± 1.18	ns

### Tumor Stage

We also stated several differences regarding the clinical presentation of the thyroid cancer in patients suffering from HT (Table 2). Among patients affected by both PTC and HT simultaneously the tumor stage (pT) was only minimally lower (average = 1.33 ± 0.73 SD), than in patients affected by PTC only (1.48 ± 0.76) (*p* = 0.065, not significant). Simillarly, we found no clear connection between pT tumor stage in FTC patients with or without HT. On the other hand, the pN status proved to be different. Surgical dissection of cervical lymph nodes was done in only 11/40 cases (27.5%) suffering from PTC and HT simultanously and in 41/190 cases (21.6%) in patients having PTC alone. The statistical revision confirmed, that in case of PTC, the nodal stage (pN) was significantly lower in the presence of HT (4/11 pN1; 34.6%) then the patients without HT (34/41 pN1; 82.9%)(*p* = 0.002). In patients with FTC and HT, cervical lymph node dissection was performed only in one out of the 3 cases, and in FTC patients without HT in 1/15 cases, not allowing further comparisons.

### BRAF and NRAS Mutational Status and HT

BRAF exon 15 V600 mutation analysis was performed in 185 thyroid carcinomas out of which mutations were detected in 89 cases (48.1%). No sequence variants further to the classical V600E mutation could be demonstrated following classical Sanger sequencing of the region. BRAF mutation was present in a significantly higher proportion of PTCs (88/158, 55.7%) than in the rest of cases presenting with other histomorphology (1/27, 3.7%) (Table 3). Within the PTC group, the classical papillary morphology was more frequently involved by the V600E mutation than the follicular variant (70/108, 64.8% vs 15/40, 36.6%, *p* = 0.0045).

**Table 3:** Correlation of BRAF V600E mutational status and histological types in thyroid carcinoma with and without coexisting HT

	**BRAF V600E**	**WT BRAF**	***p value***
**Histologic type of thyroid cancer**			
• PTC	88/158 (55.7%)	70/158 (44.3%)	
• Other than PTC (FTC, MTC, ATC)	1/27 (3.7%)	26/27 (96.3%)	<0.0001
**Histologic subtype of PTC**			
• Classical PTC	70/108 (64.8)	38/108 (35.2%)	
• Follicular variant of PTC	15/40 (37.5%)	25/40 (62.5%)	0.0045
• Other variants of PTC	3/10 (30.0%)	7/10 (70.0%)	
**Presence of HT among PTC pts**			
• HT + PTC	9/28 (32.1%)	19/28 (77.9%)	
• PTC without HT	79/130 (60.7%)	51/130 (39.3%)	<0.005
**Subtypes of PTC among HT + PTC pts**			
• Classical PTC + HT	8/18 (44.4%)	10/18 (55.6%)	
• Follicular variant of PTC + HT	0/7 (0.0%)	7/7 (100.0%)	0.0299
• Other variants of PTC + HT	1/10 (10.0%)	9/10 (90.0%)	

On the other hand, PTC associated with HT showed mutations with less frequency (9/28, 32.1%) than PTC alone (79/130, 60.7%)(*p* < 0.005). Within the PTC + HT group classical papillary morphology was associated with BRAF mutation in 8/18 cases (44.4%), any different morphology presented with much less mutation frequency (1/10, 10%). None of the cases with PTC follicular variant morphology appeared to be BRAF mutant (0/7) (Table 3).

V600E BRAF mutation could be demonstrated in 7/34 (20.6%) PTCs with lymph node metastasis (pN1) while none of the metastatic PTC + HT cases presented with the mutant-type BRAF status (0/4, 0%).

Typical oncogenic NRAS mutations of the exons 2, 3 and 4 were also tested in the majority of the cases. A low frequency of exon 2 codon 12 mutations (2/128, 1.56%) was found, all in cases with papillary histological type. Other nucleotide variants or exon 3 and 4 mutations were not be detected. None of the NRAS variants co-existed with BRAF alteration. We could not demonstrate any relation of NRAS mutant status to HT etiology, papillary morphology or multifocality of the tumor.

## Discussion

Our results - in accordance with previous studies - showed that thyroid cancer is likely to develop in thyroid glands affected by Hashimoto’s thyreoiditis and that TC coexisting with HT presents with characteristic clinico-pathological features. In association with HT we found a dominant occurrance of papillary histology, an increase in multifocality and a lower frequency of BRAF mutations.

Our tissue-level studies focussed first on the charasterictics that could be objectively judged by histology. We found no difference in either the general morphology or the IHC phenotype in PTCs with or without HT that was in agreement with earlier studies focussing on the panel composed of CK19, galectin-3 and CD56 [36]. Unlike as referred by Ma et al. (2014) the use of NGAL and claudin-1 immunostainings could be abandoned in our study. Interestingly, the HBME-1 antigen revealed the presence of neoplastic growth less sensitively in cases accompanied by HT. Using this limited IHC panel we were able to reproduce the tissue distribution and extent of thyroid cancer related to HT as published earlier.

Activation loop mutation (codon 600) of the BRAF gene leading to the amino acid exchange V600E is the most common genetic alteration in PTC [31]. It was suggested to predispose tumor multifocality, extrathyroid spreading, lymph node metastasis and advanced tumor stage [34]. The analysis of BRAF V600E sequences and the mutant BRAF protein by IHC showed a perfect correlation in our present study. However, we observed significantly lower BRAF mutation rate (*p* < 0.005) in PTCs associated with HT compared to non-HT samples. This was even more prominent in association with the follicular variant PTC (0/7 mutant cases). The mutation frequency occurring in our PTCs with HT cohort (32.1%) was similar to the one reported by Kim et al. [37]. Lymph node metastasis (pN1) was demonstrated in the minority of PTC + HT cases which all presented with a wild-type BRAF status. Metastatic non-HT PTC cases on the other hand proved to be BRAF-mutant in 7/34 (20.6%). The uncommon occurrance of mutant BRAF together with the generally higher rate of multifocality would argue against a carcinogenetic role of mutant BRAF in early tumor formation induced by HT.

Activating NRAS exon 2, 3 or 4 mutations were described as relatively rare, but significant in thyroid carcinomas occuring in approx. 10% in total and associated with adverse prognosis [38]. NRAS mutations were associated with the follicular histological type and agressive behaviour in a recent study performed by next generation sequencing [39]. Although the low number of NRAS mutations generally deteced in this collective did not allow to draw far conclusions, based on these limited data a direct carcinogenetic effect in HT-related thyroid cancer, mostly of papillary type, seems to be unlikely.

The established coexistance of HT and PTC argues for parallel pathobiological origins simultaneously involving immunological and genetic mechanisms. Subclinical-clinical forms of HT are generally the result of hyperergic autoimmune damage affecting the thyroid parenchyma. The enhanced TSH stimulus together with additional inflammatory citokines act as potential activators of aberrant cell proliferation throughout the entire parenchyma corresponding to the classical idea of „field cancerization”. However, cellular response may be regionally highly different also in regard of suspectibility to transformation. Our results also clearly demonstrate multifocality of PTC in the presence of histologically manifest HT. We definitely could separate more than one independent tumor site within the same surgical specimen in 16/40 (40.0%) of our PTC + HT cases. Although this feature is in agreement with earlier reports, we could not confirm, that multifocality or metastatic potential is associated with the mutant BRAF or NRAS genotype in cases with or without HT etiology.

For this reason, in the absence of MAPK-pathway mutations alternative genetic or biological factors should be considered in HT driven multifocal carcinogenesis. Hypothetically, basic proliferative stimuli manifested in HT act as tumorigenic in thyroid folliclular epithelial cells. One major candidate, the RET oncogene activates p21ras, which also phosphorylates wild-type BRAF, as a potent mitogen-activated protein phosphorylase. RET/PTC1 and RET/PTC3 fusion gene expression could be deteced by RT-PCR as a result of common PTC associated chromosomal aberrations [33]. Wirtschafter and collegues raised the possibility of the RET gene activation in early forms of HT. They suprisingly found that the frequency of the mentioned chromosomal aberrations in HT was as high as in manifest PTCs [40]. According to this hypothesis, the aberrant expression of the RET gene in follicular epithelial cells next to the transforming effect potentially affects the immunogenicity of the same thyreocytes. Thus, an autoimmune process with features of HT could be induced as one of the earliest signs of epithelial transformation.

The PI3K/Akt signaling pathway was implicated in the maintenance of the balance between pro- and antiapoptotic signals and in the management of the inflammatory processes [41]. The PI3K inhibitor PTEN is expressed in normal thyreocytes and in thyreocytes affected by HT, but suppressed in transformed epithelial cells of PTC. In samples involved by both HT and PTC intermediate levels of PTEN expression was reported [37], arguing for an augmented PI3K activity suppressing proapoptotic signals. Further, the p53 homolog p63 protein –among others responsible for a stem cell-like phenotype- may also interplay with HT-related thyroid carcinogenesis. Alternative splicing results in a smaller, truncated p63 molecule, which is a competitive inhibitor of the tumor supressor p53. In contrast to the normal epithelium, focal p63 expression was reported in PTC, especially in the classical papillary type and in most cases of HT [42].

Chronic immune-activation or autoimmune disease related carcinogenesis is a well known mechanism generally associated with slow transformation rates and a generally indolent potential in e.g. GI tract, liver, skin or lymphoid neoplasias [43]. In line with these another interesting question is why PTCs accompanied with HT generally show a relatively benign behavior. Marotta et al. [44] observed that lymphocytic inflitration of HT is protective against PTC progression. Similarly, Kim et al. [45] found that HT-negative papillary TC was associated with an aggressive disease. These data together with our current results support a molecular biology different of the MAP kinase pathway with limited progressive capacity in the background of these lesions. However, special care of patients having syptoms due to their autoimmune pathology should not be ignored when considering progressive disease. As also stated by the present study, the pathologic stage of the tumor (diameter) proved to be independent of HT in earlier reports, however, the HT had a „protective” effect against extrathyroid manifestation and nodal involvement [46]. In our understanding the presence of enlarged lymph nodes greatly affect clinical decisions. As HT is frequently complicated by lymphadenomegaly due to persistent immune activation and lymphatic hyperplasia, regional lymph nodes are subjects of careful clinical follow-up. Thus, the regular control of thyroid gland and lymph node status in HT patients may significantly contribute to the early detection and surgical treatment of thyroid carcinoma.

The present data, following the earlier reports confirm that HT can be regarded as a pre-condition for PTC highlighted by specific biological features other than BRAF or NRAS activating mutations. Thanks to the effective long term clinical care of the underlying disease, PTC can be recognised earlier which also contributes to the prevention of advanced disease and metastatic dissemination.

## Abbreviations

ATC: anaplastic thyroid carcinoma
UD CC: University of Debrecen Clinical Centre
HT: Hashimoto’s thyreoiditis
FTC: follicular thyroid carcinoma
MF: multifocal
MTC: medullary thyroid carcinoma
PTC: papillary thyroid carcinoma
TSH: thyroid stimulating hormone
UF: unifocal
